# First record of *Orobdella kawakatsuorum* (Hirudinida: Arhynchobdellida: Erpobdelliformes) from Kunashir Island, Kuril Islands

**DOI:** 10.3897/BDJ.2.e1058

**Published:** 2014-02-17

**Authors:** Takafumi Nakano, Konstantin B. Gongalsky

**Affiliations:** † Department of Zoology, Graduate School of Science, Kyoto University, Kyoto, Japan; ‡ A.N. Severtsov Institute of Ecology and Evolution, Russian Academy of Sciences, Moscow, Russia

**Keywords:** Hirudinida, *Orobdella kawakatsuorum*, geographical record, ND1, Kuril Islands

## Abstract

Specimens of the genus *Orobdella* Oka, 1895 from Kunashir Island, the Kuril Islands, are identified as *Orobdella kawakatsuorum* Richardson, 1975. Mitochondrial tRNA^Leu^ and ND1 data confirm the species identification of the Kunashir specimens. This is the first record of the genus *Orobdella* from the Kuril Islands.

## Introduction

The genus *Orobdella* Oka, 1895 is a terrestrial macrophagous leech taxon that contains 11 described species from Japan, Korea, and Taiwan (Nakano 2012b, Nakano and Lai 2012, Nakano and Seo 2012). *Orobdella whitmani* Oka, 1895 was reported from Primorsky Krai in the continental part of the Russian Far East (Gilyarov and Perel 1971, Gilyarov et al. 1969, Gongalsky 2007, Lukin 1976). However, the identification of the Russian specimens is doubtful (Nakano 2012a). The northernmost distributional limits of this genus have been reported as Primorsky Krai of the Russian Far East, and the Shiretoko Peninsula of Hokkaido where two species, *Orobdella kawakatsuorum* Richardson, 1975 and *Orobdella koikei* Nakano, 2012, have been recorded (Nakano 2012a).

The Kuril Islands are a long archipelago off the coast of the Russian Far East located between the Kamchatka Peninsula and Hokkaido. Members of *Orobdella* have never been recorded from the Kuril Islands, although the southern part of the islands lies close to Hokkaido. Recently, several *Orobdella* specimens were collected on Kunashir Island by the second author during a survey of soil fauna on the island. Based on morphological examination of the specimens, the identification and brief description of the Kunashir *Orobdella* are herein presented. In addition, mitochondrial tRNA^Leu^ and ND1 sequence data of the specimens are presented as confirmation of species identity based on their morphological characteristics.

## Materials and methods

**Figure 1.**
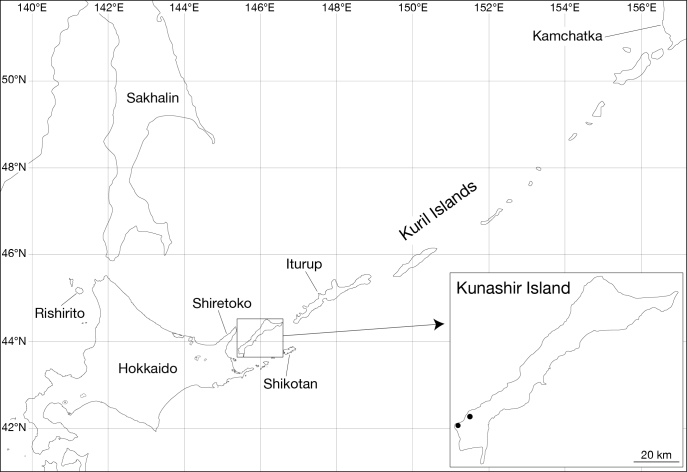
Map showing the Kuril Islands and adjacent areas. Filled circles indicate collection localities of the specimens examined in this study.

Leeches were collected from Kunashir Island in the Kuril Islands (Fig. 1). The specimens were preserved in 95% ethanol in the field. In the laboratory, botryoidal tissue was taken from the posterior part of the body around the caudal sucker of each specimen for DNA extraction, and the rest of the bodies were re-fixed in 10% formalin for dissection and preserved in 70% ethanol. Examination, dissection, and drawings of the specimens were done under a stereoscopic microscope equipped with a drawing tube (Leica M125). Specimens used in this study have been deposited in the Zoological Collection of Kyoto University (KUZ).

The numbering convention is based on the system adopted by Moore (1927): body somites are denoted by Roman numerals, and the annuli in each somite are given an alphanumeric designation.

Sequences of mitochondrial tRNA^Leu^ and ND1 (tRNA^Leu^–ND1) were determined for 4 specimens of *Orobdella kawakatsuorum* and *Orobdella koikei*, and one specimen of *Orobdella whitmani* Oka, 1895, in addition to the two specimens from Kunashir Island (Table 1). The extraction of genomic DNA and DNA sequencing methods followed Nakano (2012a). For obtaining sequences of tRNA^Leu^–ND1, the procedure was modified as follows: the primer set was LND300 and HND1932 (Light and Siddall 1999); the PCR reaction mixture was heated to 94 °C for 5 min, followed by 40 cycles at 94 °C (10 s), 55 °C (20 s), and 72 °C (39 s), and a final extension at 72 °C for 6 min; the sequencing reaction mixture was incubated at 96 °C for 2 min, followed by 40 cycles at 96 °C (10 s), 50 °C (5 s), and 60 °C (39 s). Newly obtained sequences have been deposited with the International Nucleotide Sequence Database Collaboration (INSDC; Table 1).

The length of the obtained tRNA^Leu^–ND1 was 629 bp for *Orobdella whitmani* (KUZ Z45) and 630 bp for the other specimens. These sequences were aligned using MAFFT FFT-NS-2 (Katoh et al. 2005). The length of the aligned sequences was 630 bp. A gap was inserted in the tRNA^Leu^ part of the sequence of *Orobdella whitmani*. Genetic distances of the obtained sequences were calculated by Kimura 2-parameter (K2P) correction (Kimura 1980), and then a neighbor-joining tree was constructed with nonparametric bootstrapping based on 1000 replicates using MEGA 5 (Tamura et al. 2011).

## Taxon treatments

### Orobdella kawakatsuorum

Richardson, 1975

urn:lsid:zoobank.org:act:BE8762C7-7AAB-49FD-9488-E8F32E324A27

http://species-id.net/wiki/Orobdella_kawakatsuorum

#### Materials

- **Type status:** Other material. **Occurrence:** catalogNumber: KUZ Z675; individualCount: 1; sex: hermaphrodite; **Location:** island: Kunashir Island; verbatimLocality: near Ivanovsky cordon of Kurilsky Nature Reserve, 600 m from the Sea of Okhotsk, Kunashir Island; decimalLatitude: 43.839933N; decimalLongitude: 145.412833E; **Identification:** identifiedBy: Takafumi Nakano; **Event:** eventDate: 2012-08-23; habitat: Oak (*Quercus crispula*) forest with bamboo (*Sasa* sp.) and lianas (*Hydrangea paniculata*, *Vitis coignetiae*), forest canopy density 60%, grass cover density 100%, litter depth up to 15 cm; **Record Level:** institutionCode: KUZ
- **Type status:** Other material. **Occurrence:** catalogNumber: KUZ Z676; individualCount: 1; sex: hermaphrodite; **Location:** island: Kunashir Island; verbatimLocality: near Ozernyi cordon of Kurilsky Nature Reserver, on the eastern slope of Golovnin Volcano caldera, 1 km from the Sea of Okhotsk, Kunashir Island; decimalLatitude: 43.875333N; decimalLongitude: 145.476617E; **Identification:** identifiedBy: Takafumi Nakano; **Event:** eventDate: 2012-08-26; habitat: Fir (*Abies sachalinensis*) forest with birch (*Betula platyphylla*) and oak (*Quercus crispula*) and bamboo (*Sasa* sp.), forest canopy density 70%, grass cover density 80%; **Record Level:** institutionCode: KUZ

#### Description

Body firm, muscular, elongated, with constant width in caudal direction, dorsoventrally compressed, BL 23.8–32.5 mm, BW 3.7–4.9 mm (Fig. 2). Caudal sucker elliptic, minor axis 1.0–1.6 mm, major axis 1.9–2.7 mm (Fig. 2b). Somite I completely merged with prostomium. Somites II, III uniannulate. Somites IV, V biannulate. Somites VI, VII triannulate. Somites VIII–XXV quadrannulate (Fig. 3a). Somite XXVI triannulate. Somite XXVII uniannulate; anus behind it. Eyes in 3 pairs, first pair dorsally on posterior margin of II, second and third pairs dorsolaterally on posterior margin of V (a1 + a2). Nephridiopores in 17 pairs, 1 each situated ventrally at posterior margin of a1 of each somites in VIII–XXIV (Fig. 3a).

Pharynx reaching to XIV b5/b6 (Fig. 3b). Crop reaching to XX b5–XX/XXI (Fig. 3b). Gastropore in furrow of XIII a1/a2 (Fig. 3a, c). Gastroporal duct tubular, joining with crop in XIV b5/b6 (Fig. 3b). Intestine reaching to XXV a1/a2–b5/b6. Rectum descending to anus.

Male gonopore at anterior margin of XI b6 (Fig. 3a). Female gonopore in furrow of XIII a1/a2 (Fig. 3c). Gonopores separated by 6 annuli (Fig. 3a). Testisacs undeveloped, undetectable. Paired epididymides in XVI b5/b6–XVII a1/a2, occupying 2 annuli (Fig. 3d). Ejaculatory ducts in XI b5 to XVI b5/b6, nearly straight. Atrial cornua undeveloped. Atrium globular, in XI b6. Paired ovisacs globular, 1 each in XIII a2 and b5. Oviducts short, both oviducts converging into common oviduct in XIII a2. Common oviduct directly descending to female gonopore.

#### Distribution

*Orobdella kawakatsuorum* is distributed in Hokkaido, Japan, and its peripheral islands and inhabited in mountainous regions of these islands (Nakano 2012a). The present specimens have extended the known distributional range of the species north to include the southern tip of the Kuril Islands.

#### Genetic data

**Figure 4.**
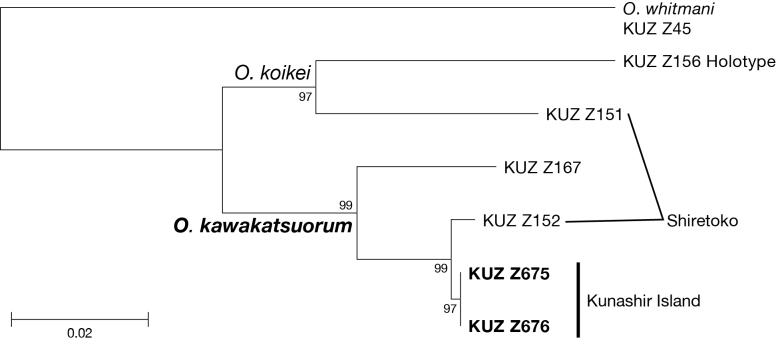
The neighbor-joining tree of 630 bp of tRNA^Leu^ and ND1. The numbers at the nodes represent the bootstrap values.

The obtained neighbor-joining tree (Fig. 4) showed that two *Orobdella* specimens from Kunashir Island (KUZ Z675, Z676) formed a monophyletic lineage with the individual of *Orobdella kawakatsuorum* from Shiretoko, Hokkaido (KUZ Z152). No difference between the tRNA^Leu^–ND1 sequences from the Kunashir specimens. The K2P distance was detected between these two specimens (KUZ Z675, Z676) and that from Shiretoko (KUZ Z152) was 0.5%.

#### Taxon discussion

Two specimens of *Orobdella* from Kunashir Island clearly belong to *Orobdella kawakatsuorum* based on the following characteristics: male gonopore in the anterior margin of XI b6, female gonopore in the furrow of XIII a1/a2, 6 annuli between gonopores, and epididymides occupying 2 annuli. According to Nakano (2012a), *Orobdella kawakatsuorum* grows to ca. 10 cm length. However, the body length of the Kunashir specimens is only ca. 3 cm. Since they have undeveloped male atria and undetectable testisacs, they were considered immature individuals. As noted in the Introduction, two quadrannulate species of *Orobdella*, *Orobdella kawakatsuorum* and *Orobdella koikei*, are distributed in Hokkaido. *Orobdella koikei* is the closest congener of *Orobdella kawakatsuorum* according to the recent molecular phylogenetic study and the smallest species among the known species of *Orobdella* (Nakano 2012a). The body length of the known mature leeches of *Orobdella koikei* is less than 4 cm. Therefore, based only on their body length, a possibility exists that the Kunashir specimens might be misidentified as *Orobdella koikei*. However, *Orobdella kawakatsuorum*, as well as the present specimens, are clearly distinguished from *Orobdella koikei* in the characteristics mentioned above: the latter possesses 1/2 + 4 + 1/2 annuli between gonopores (male gonopore in the middle of XI b6, female gonopore in the middle of XIII a1), and the epididymides occupy 9–12 annuli. Our tRNA^Leu^–ND1 data provided additional confirmation that the *Orobdella* leeches from Kunashir Island were identified correctly as *Orobdella kawakatsuorum*.

*Orobdella kawakatsuorum* was collected from Rishirito Island (Nakano 2012a), which is located ca. 20 km away from Hokkaido (Fig. 1). Additionally, Kunashir Island lies offshore of Hokkaido. Thus, anticipating that *Orobdella* leeches might occur on the island was not difficult. Our findings suggest that *Orobdella* species may also be present in the southern part of the Kuril Islands, e.g., Iturup Island and Shikotan Island. Further faunal surveys should be conducted not only in the South Kurils, but also in the northern part of the Kuril Islands, as well as the Kamchatka Peninsula, to fully reveal the northern distributional limit of the genus *Orobdella*.

## Supplementary Material

XML Treatment for Orobdella kawakatsuorum

## Figures and Tables

**Figure 2a.**
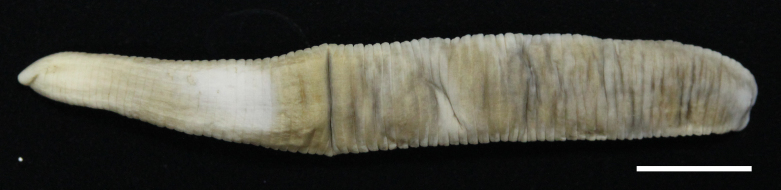
dorsal view. Scale bar: 5 mm.

**Figure 2b.**
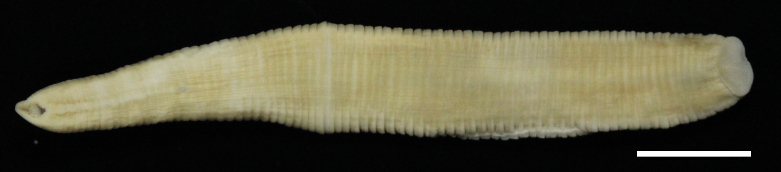
ventral view. Scale bar: 5 mm.

**Figure 3a.**
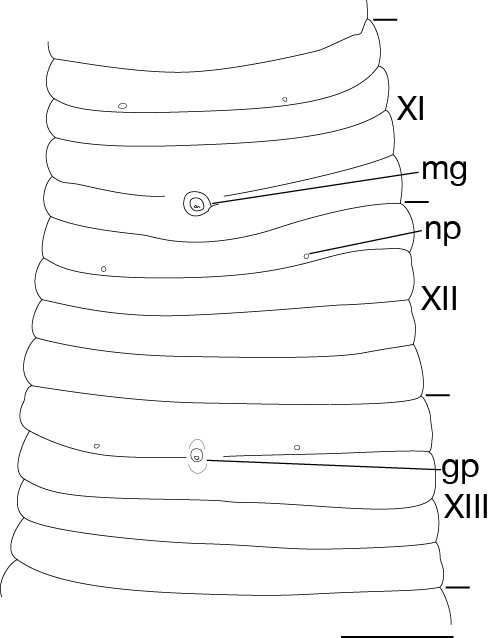
ventral view of somites XI–XIII. Scale bar: 1 mm.

**Figure 3b.**
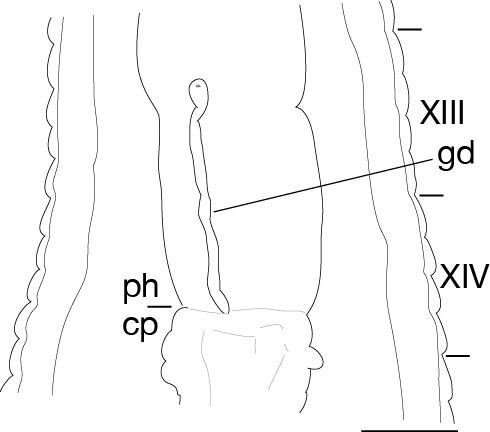
ventral view of the gastroporal duct. Scale bar: 1 mm.

**Figure 3c.**
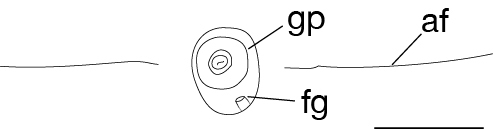
ventral view of the gastropore and female gonopore. Scale bar: 0.25 mm.

**Figure 3d.**
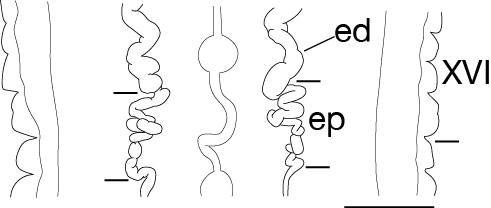
dorsal view of sperm ducts and ventral nervous system. Scale bar: 1 mm.

**Table 1.** Samples used for the DNA analysis, with the information on vouchers and INSDC accession numbers.

Voucher	Locality	tRNA^Leu^ and ND1
*Orobdella kawakatsuorum*		
KUZ Z675	Kunashir Island, the Kuril Islands	AB893606
KUZ Z676	Kunashir Island, the Kuril Islands	AB893607
KUZ Z152	Mt. Rausudake, Shiretoko, Hokkaido	AB893605
KUZ Z167	Sapporo, Hokkaido (app. 5 km far from type locality)	AB828561
*Orobdella koikei*		
KUZ Z151	Mt. Rausudake, Shiretoko, Hokkaido	AB893604
KUZ Z156 (holotype)	Sounkyo, Hokkaido	AB828560
*Orobdella whitmani*		
KUZ Z45	Mt. Kinkazan, Gifu, Honshu (type locality)	AB828556
